# COVID-19 and the alarming rise of “black fungus” (mucormycosis) infection

**DOI:** 10.11604/pamj.2022.41.318.30147

**Published:** 2022-04-20

**Authors:** Rumi Khajotia

**Affiliations:** 1 International Medical University, Hospital Tuanku Ja´afar, Seremban, Malaysia

**Keywords:** COVID-19 infection, fungal infections, mucormycosis, pulmonary infections

## Abstract

COVID-19 which first raised its deadly head in December 2019, has now engulfed the entire planet with its fire and fury. Mankind has been literally held to ransom by this micro-beast which has caused so much pain, sorrow and suffering, leaving behind scores of people dead and millions sick and gasping for air (quite literally!) The whole world is in disarray since the past 16 months, and now a new deadly superadded fungal infection has appeared in COVID-19 patients, in parts of the Indian subcontinent; namely mucormycosis, the deadly “black fungus.” This persistent, unrelenting fungal infection which is relatively resistant to conventional anti-fungal treatment, sometimes requires radical, extensive surgical intervention in order to stem the spread of infection to vital organs such as the heart, brain, orbital spaces and spleen. mucormycosis has been increasingly seen to occur in COVID-19 patients who are immunocompromised and have uncontrolled diabetes mellitus as a comorbidity. Commonly seen forms of mucormycosis in COVID-19 patients include, Rhinocerebral mucormycosis and Pulmonary mucormycosis, with some patients also developing the cutaneous form, while some manifesting the more serious disseminated form of mucormycosis.

## Commentary

Mucormycosis (popularly known as “black fungus”) is a rapidly spreading, unrelenting fungal infection which can be devastating in its intensity and reach, as is apparent in recent months, in patients combating COVID-19 infection. While more than two-thirds of the reported cases have been from the Indian sub-continent, the vast African continent too needs to be alerted about the potential devastation mucormycosis can cause in immunocompromised COVID-19 patients, who also have concomitant uncontrolled diabetes mellitus as a significant comorbidity. Mucormycosis, also known as zygomycosis is a fairly rare but devastating fungal infection, caused by a group of molds called mucormycetes. These molds are ubiquitous in our environment and live mostly in moist soil and decaying organic matter, such as compost piles, rotten wood and leaves [1,2]. Fungi that most commonly cause mucormycosis include, Mucor species, *Cunninghamella bertholletiae, Syncephalastrum* species, *Rhizopus* species, Lichtheimia and the Apophysomyces species [1].

**Clinical characteristics**: mucormycosis mainly affects people who have comorbid conditions such as uncontrolled diabetes mellitus, and a weakened immune response due to the intake of medications such as corticosteroids, which lower the body´s response to infections. Other major risk factors for contracting mucormycosis include prolonged ICU stay, voriconazole therapy and underlying malignancies. Mucormycosis mainly affects the nasal and paranasal sinuses and the lungs. The mode of entry is by inhaling fungal spores from the atmosphere. It can also gain entry through the skin following an injury, a burn, a cut or an abrasion.

Mucormycosis can be classified into the following categories.

**Rhinocerebral mucormycosis**: it is a fulminanting infection in the sinuses that has the potential to spread to the brain. This form of mucormycosis is most common in people with uncontrolled diabetes mellitus and in people who have been immunocompromised in any way, such as due to intake of corticosteroids or immunosuppressants, following organ transplant [3,4]. This form of mucormycosis has now become a cause of growing concern, as it has also been observed in patients following COVID-19 infection, in some parts of the world.

**Pulmonary (lung) mucormycosis** is a very common type of mucormycosis. It is mainly seen in immunocompromised patients such as those who have had an organ transplant or those with cancer.

**Disseminated mucormycosis** occurs when the infection gets disseminated through the bloodstream and affects distant organs such as the heart, brain, skin and spleen.

**Gastrointestinal mucormycosis** is a condition more frequently observed in young children; especially in premature and low birth weight infants less than 1 month of age, who have had medications that lower the body´s immunity to fight disease [5,6].

**Cutaneous mucormycosis** occurs when the fungi gains entry following a break in the skin as a result of an abrasion, cuts or severe burns. This is the most common form of mucormycosis among people who do not have a compromised immunity. Symptoms of mucormycosis manifest depending on the point-of-entry of the fungus into the human body, and the site of growth and dissemination [7,8]. Most commonly, symptoms of rhinocerebral mucormycosis include facial swelling, mostly on one side of the face, severe headache, fever, congested nose and pain in the maxillary and frontal sinuses. One characteristic feature of mucormycosis is the presence of “black lesions” on the nasal bridge or the hard palate, that quickly become severe and fulminant, giving it the name “black fungus.” Rhinocerebral mucormycosis may be accompanied by pulmonary mucormycosis with symptoms such as cough, fever, chest pain and breathlessness.

**Incidence of mucormycosis in COVID-19 patients**: the rising incidence of mucormycosis which has an overall mortality rate of 50% in patients with COVID-19 infection, is strongly presumed to be due to the indiscriminate use of corticosteroids, combined with uncontrolled diabetes mellitus in severe and critically ill Covid-19 patients. Steroids reduce inflammation in the lungs in COVID-19 and appear to help stop some of the damage that can happen during a cytokine storm, when the body's immune system goes into overdrive, to ward off the coronavirus. But steroids also reduce the body´s immunity and elevate blood sugar levels in both diabetic and non-diabetic COVID-19 patients, which is now believed to be the triggering factor for the development of mucormycosis in these patients. As a result, a rising incidence of cases of rhino-orbital mucormycosis in COVID-19 patients have been observed over the last many months, on the Indian subcontinent. Six cases of COVID-19 infection with rhino-orbital mucormycosis were reported recently at a single institution [9]. Of these, one patient had concurrent COVID-19 and mucormycosis at the time of admission, while five other patients developed mucormycosis during course of treatment with systemic corticosteroids for COVID-19 infection. Another study [10], reported 10 cases of orbital mucormycosis with concomitant COVID-19 infection. These patients presented with clinical features of both orbital mucormycosis and COVID-19 on screening. All these patients were known diabetics, with diabetic ketoacidosis (DKA) being present in four of these patients at the time of admission. Later, five patients developed diabetic ketoacidosis after the initiation of corticosteroid therapy for the COVID-19 infection. All patients in this group received intravenous dexamethasone, possibly to combat an impending cytokine storm. All patients received liposomal Amphotericin B (LAmB) for mucormycosis. Four patients died within 1 month of the diagnosis, while five patients had permanent loss of vision. Many other instances of rhinocerebral mucormycosis too have been reported, with some patients needing removal of the entire upper jaw, and in some cases eyeball enucleation, to prevent the fungus from spreading to the brain. All these patients had concurrent diabetes mellitus and were on intravenous corticosteroids, in the course of treatment for their COVID-19 infection. Consequently, rampant use of corticosteroids coupled with uncontrolled diabetes mellitus as a comorbid condition, is presumed to be one of the main factors for the increasing incidence of mucormycosis, in patients with COVID-19 infection.

**Management and treatment options**: in order to prevent mucormycosis it is recommended that blood glucose levels be kept under control through constant monitoring, even following post-COVID discharge, in diabetic patients. Judicious use of corticosteroids in correct dosage forms and duration is essential for prevention of this fungal disease in COVID-19 patients. It is also recommended that only clean sterile water be used in oxygen humidifiers, and antibiotics and antifungal medication be used in correct dosage forms and for the appropriate duration. Mucormycosis is a serious, unrelenting, persistent infection that needs to be treated with prescription antifungal medication such as liposomal amphotericin B (LAmB), lipid complex amphotericin B, posaconazole, or isavuconazole for at least six weeks. These medications are administered intravenously (amphotericin B, posaconazole, isavuconazole) or orally (posaconazole, isavuconazole). During treatment for mucormycosis, it is recommended to insert a central catheter line for maintaining adequate systemic hydration, and for administration of the appropriate medication. The recommended starting dose of liposomal amphotericin B in mucormycosis is 3-5mg/kg/day, given intravenously over a 30-60 minutes period. The duration of therapy is usually determined on an individual case-to-case basis, but the normal duration of treatment is usually 6-8 weeks. A longer duration of treatment may be required in patients with deep-seated bone, palate and orbital infections, those on prolonged chemotherapy and in those with severe neutropenia.

Prior to starting treatment with amphotericin B, a test dose of 1mg must be given intravenously over 10 minutes, and the patient must be assessed over the next 30 minutes for any severe allergic or anaphylactic reactions. If the medication is tolerated well, it can be continued. Hopefully, prompt use of the above medications, compounded with a good control of blood sugar levels and a more judicious use of corticosteroids in COVID-19 patients, will help in containing the rising incidence of mucormycosis in these patients. It should be noted that medications such as fluconazole, voriconazole, and echinocandins are ineffective against fungi that cause mucormycosis. Sadly, since mucormycosis is an unrelenting disease, more often than not, it entails radical surgery to cut away the infected tissue. This may include entire jaw removal or enucleation of an eyeball, in order to prevent further spread of the fungal infection into the brain, or disseminate to organs such as the heart, spleen and other vital organs.

In conclusion, it appears that the spread of mucormycosis in COVID-19 patients is assuming alarming proportions in some parts of the world, namely, the Indian subcontinent. This is mainly seen in patients with uncontrolled diabetes mellitus who are on corticosteroids and other immunomodulating agents. Clinicians in Africa therefore need to stay alert to the possibility of this unrelenting fungal infection presenting in COVID-19 patients here. A high-index of clinical suspicion in patients presenting with typical symptoms and signs, will go a long way in promptly diagnosing and treating this potentially fatal infection.
